# *Pyruvate Kinase M2* Expression: A Potential Metabolic Biomarker to Differentiate Endometrial Precancer and Cancer that is Associated with Poor Outcomes in Endometrial Carcinoma

**DOI:** 10.3390/ijerph16234589

**Published:** 2019-11-20

**Authors:** Yu-Ju Lai, Yu-Ching Chou, Yi-Jia Lin, Mu-Hsien Yu, Yu-Che Ou, Po-Wei Chu, Chia-Chun Wu, Yu-Chi Wang, Tai-Kuang Chao

**Affiliations:** 1 Department of Obstetrics and Gynecology, Tri-Service General Hospital, National Defense Medical Center, Taipei 114, Taiwan; yuru220@hotmail.com (Y.-J.L.); hsienhui@ms15.hinet.net (M.-H.Y.); powei1113@gmail.com (P.-W.C.); yuchitsgh@gmail.com (Y.-C.W.); 2 Department of Obstetrics and Gynecology, Tri-Service General Hospital, Magong City 88056, Penghu Branch, Taiwan; 3 School of Public Health, National Defense Medical Center, Taipei 114, Taiwan; trishow@mail.ndmctsgh.edu.tw; 4 Department of Pathology, Tri-Service General Hospital, National Defense Medical Center, Taipei 114, Taiwan; b93401052@ntu.edu.tw; 5 Department of Obstetrics and Gynecology, Chiayi Chang Gung Memorial Hospital, Chiayi 600, Taiwan; tedycou@gmail.com; 6 Department of Obstetrics and Gynecology, Kaohsiung Chang Gung Memorial Hospital and Chang Gung University College of Medicine, Kaohsiung 800, Taiwan; 7 Department of Orthopedics, Tri-Service General Hospital, National Defense Medical Center, Taipei 114, Taiwan; doc20281@gmail.com

**Keywords:** pyruvate kinase M2, endometrial hyperplasia, endometrial carcinoma

## Abstract

Background: Pyruvate kinase M2 (PKM2) is a regulator of the processes of glycolysis and oxidative phosphorylation, but the roles that it plays in endometrial cancer remain largely unknown. This study evaluated the PKM2 expression in normal endometrium, endometrial hyperplasia, and endometrial carcinoma, and its prognostic value was investigated in endometrial carcinoma patients. Methods: A hospital-based retrospective review was conducted to examine the immunohistochemical PKM2 distribution in 206 endometrium samples from biopsies or hysterectomies. The immunoreactivity of PKM2 was divided into groups of low and high scores according to the extent and intensity of staining. Results: Intense cytoplasmic staining was observed for the PKM2 protein in malignant endometrial lesions. A high PKM2 score was observed in many endometrial carcinoma samples (50.0%), but there was a low percentage in endometrial atypical hyperplasia (12.5%). High PKM2 expression was not found in the normal endometrium (0.0%) nor endometrial hyperplasia without atypia (0.0%). The PKM2 protein score was significantly higher in endometrial carcinoma samples than premalignant endometrial lesions (*p* < 0.001). Notably, higher PKM2 scores in cases of endometrial carcinoma correlated with poor overall survival (*p* = 0.006), and the hazard ratio for death was 3.40 (95% confidence interval, 1.35–8.56). Conclusions: Our results indicate that the prevalence of PKM2^high^ tumor cells in endometrial carcinoma is significantly associated with worse prognostic factors and favors a poor prognosis. The expression of PKM2 is also a potential histopathological biomarker for use in the differential diagnosis of malignant and premalignant endometrial lesions.

## 1. Introduction

Endometrial carcinoma (EC) is one of the most common gynecological cancers worldwide [1]. According to statistics from the American Cancer Society, there were about 63,230 new cases of EC in 2018, and 11,350 women died from it. About 67% of EC cases can be diagnosed at an early stage with the disease localized to the uterus. However, other cases may be diagnosed at advanced stages with early metastasis and a poor survival rate. 

Endometrial hyperplasia (EH) is clinically significant because of the associated risk of progression to endometrioid EC, and atypical forms of EH are considered as premalignant lesions [2]. There are major challenges in the differential diagnosis between atypical hyperplasia (AH) and well-differentiated EC due to poor reproducibility for cytological atypia [3,4]. Paradoxically, more atypical features may be observed in cases of AH than EC, and some well-differentiated ECs exhibit quite unremarkable cytology [5]. 

Several immunohistochemical biomarkers have been explored as diagnostic adjuncts for the pathological diagnosis of endometrial lesions. However, no efficient immunohistochemical biomarker candidate has been found thus far to reliably satisfy the requirements and reproducibly distinguish between premalignant and malignant endometrium [6]. There is great need for new biomarkers to achieve better differential pathological diagnoses of malignant and premalignant endometrial lesions. 

The hallmarks of cancer are composed of six biological capabilities that are acquired during the multiple steps of development of human tumors [7]. Progress in the last decade has revealed two emerging hallmarks of this list: reprogramming of energy metabolism and evading immune destruction. Alterations in metabolites can accompany cancer-associated metabolic reprogramming and profoundly affect gene expression, cellular differentiation, and the tumor’s microenvironment. Glucose and glutamine are two principal nutrients that support survival and biosynthesis in mammalian cells [8].

Pyruvate kinase M2 (PKM2) is a key energy enzyme that is involved in regulating cell glycolysis metabolism. In mammals, PK has four isoforms: PKL, PKR, PKM1, and PKM2. In the final step of glycolysis, it catalyzes the conversion of phosphoenolpyruvate (PEP) and ADP into pyruvate and ATP [9]. Aerobic glycolysis commonly occurs in most cancer cells and allows them to produce energy. This is followed by lactic acid fermentation, even in the presence of oxygen, which is known as the “Warburg effect” [9,10]. Recently, studies have demonstrated that PKM2 is also a major isoform that is expressed in cancer cells [10,11].

We previously found that PKM2 is overexpressed in cases of ovarian cancer, and inhibiting PKM2 using shikonin resulted in inhibited growth of ovarian cancer cells [12]. The expression profile of PKM2 in endometrial lesions is currently unclear. Aerobic glycolysis in cancer cells requires PKM2 and is a hallmark of cancer metabolism as well as a major source of energy that is essential for the growth and survival of cancer cells. Thus, it may be a potential biomarker to identify EC from premalignancies. The aim of this study was to determine the potential of PKM2 distribution as a single biomarker for differential diagnosis. We also investigated the clinical significance of the distribution of PKM2 in EC tissues.

## 2. Materials and Methods 

### 2.1. Tissue Microarray

Tissues from Chinese patients embedded in Paraffin wax were retrospectively retrieved from the Department of Pathology at Tri-Service General Hospital, and tissue microarray slides were constructed as described previously [13]. A total of 206 endometrial specimens were collected by following the 1994 guidelines for EH classification from the World Health Organization (WHO) [14] and the 2009 staging system classifications of the International Federation of Gynecology and Obstetrics (FIGO) [15]. Stage IA is defined as tumors in the endometrium only or less than halfway to the endometrium. Stage IB is defined as tumors spread halfway or more into the myometrium. Stage II is defined as tumors involved in the cervical stroma but not beyond the uterus. Stage III is defined as tumors involved in the serosa, adnexa, vagina, or parametrium. Stage IV is defined by tumors that have invaded the bladder and/or bowel mucosa, distant metastases, or inguinal lymph nodes. 

All the selected samples were obtained with informed consent, and this study was approved by the Institutional Review Board of Tri-Service General Hospital. The tissue microarrays consisted of 206 samples and included 30 normal endometrium, 36 EH, 32 AH, and 108 EC samples. The EC samples were obtained from women who underwent a standard staging operation to treat a known endometrial malignancy, including hysterectomy and pelvic/para-aortic lymphadenectomy. For EC specimens, only cases with completed guideline-recommended treatments were included. Although type I EC is related to AH progression, we included some type II carcinoma specimens in the analysis to identify a possible relationship. The tissue microarrays comprised 108 samples of EC, but 23 cases were lost to follow-up and did not have survival data. The remaining 85 EC cases included 64 endometrioid adenocarcinoma (EmAC) samples, 11 serous carcinoma (SC) samples, 8 clear cell carcinoma (CC) samples, and 2 mucinous carcinoma (MC) samples. All of these cases received surgical hysterectomy after tissue confirmation. Two pathologists screened the histological sections and selected areas of representative tumor cells, and one tissue core (2 mm in diameter) was then taken from each of the representative tumor samples and placed in a new recipient paraffin block for immunohistochemistry staining.

This study was approved by the Institutional Review Board of the Tri-Service General Hospital (TSGHIRB No: 2-101-05-041 and 2-103-05-144 and 2-104-05-022) and obtained written consent to approve this consent procedure. Informed consent was obtained from all patients.

### 2.2. Immunohistochemistry Stain

The tissue microarray sections were dewaxed in xylene, rehydrated in alcohol, and immersed in 3% hydrogen peroxide for 10 min to suppress the activity of endogenous peroxidase. Antigen retrieval was carried out by heating each section to 100 °C for 30 min in 0.01 M sodium citrate buffer (pH 6.0). The sections were rinsed three times (5 min each wash) in phosphate-buffered saline (PBS) and then incubated for 1 h at room temperature with a 1:200 dilution of rabbit polyclonal anti-human PKM2 antibody (Abcam, cat # ab85542, Cambridge, UK) in PBS. The sections were washed three times (5 min each wash) in PBS, followed by incubation with horseradish peroxidase-labeled immunoglobulin (Dako, Carpinteria, CA, USA) for 1 h at room temperature. 

The sections were washed three times again, and the peroxidase activity was visualized using a solution of diaminobenzidine (DAB) at room temperature. All tissue microarray slides were examined in terms of immunoreactivity and histological appearance and independently scored concurrently by two of the authors. The immunoreactivity was graded arbitrarily and semiquantitatively based on the intensity and percentage of staining on the tissue microarray slides, as described previously [16,17]. The PKM2 intensity in individual tumor cells was scored as 0 (no staining), 1+ (weak intensity), 2+ (moderate intensity), or 3+ (strongest intensity). The staining of cells was scored as negative (<30% of the area was positive), 1+ (30%–60% of the area was positive), or 2+ (>60% of the area was positive). 

The absolute value of the proportion of cells at each intensity level was multiplied by the corresponding intensity value, and then the products were added to obtain immunostaining scores of 0+, 1+, 2+, 3+, 4+, or 6+. PKM2^low^ was defined as a score of 0+ or 1+, and PKM2^high^ was defined as a score of 2+, 3+, 4+, or 6+. Slides incubated with nonimmune serum instead of the primary antibody were used as negative controls. 

### 2.3. Statistical Analysis

Patients’ clinical data were retrieved from hospital patient files. All values are expressed as the mean ± standard error of the mean (SEM) and as percentages. An analysis of variance (ANOVA) and chi-squared tests were performed to compare the PKM2 levels of different groups of normal endometrium, EH, AH, and EC samples. Associations between PKM2 levels and clinicopathological characteristics were identified using the chi-squared test or Fisher’s exact test. The overall patient survival time was monitored at the hospital. 

Cox regression analysis was used to assess the data. Kaplan–Meier survival curves were compared using the log-rank test. Two-sided p-values <0.05 were considered significant. All analyses were performed using SPSS for IBM, version 21 (IBM Corp., Armonk, NY, USA).

## 3. Results

**Figure 1:**
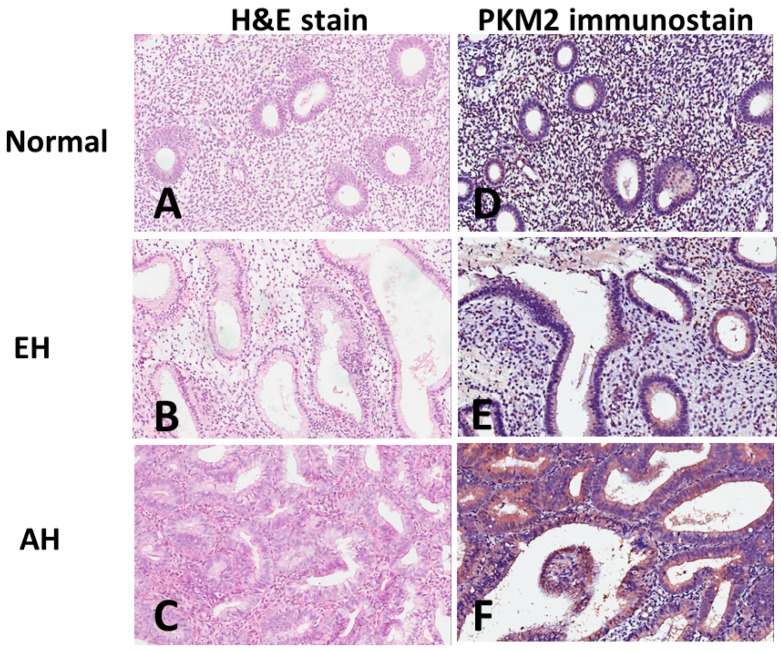
Examples of hematoxylin and eosin (H&E) staining of (**A**) normal endometrium, (**B**) endometrial hyperplasia without atypia, (**C**) atypical hyperplasia and pyruvate kinase M2 (PKM2) immunostaining of (**D**) normal endometrium, (**E**) endometrial hyperplasia without atypia, (**F**) atypical hyperplasia.

**Figure 2:**
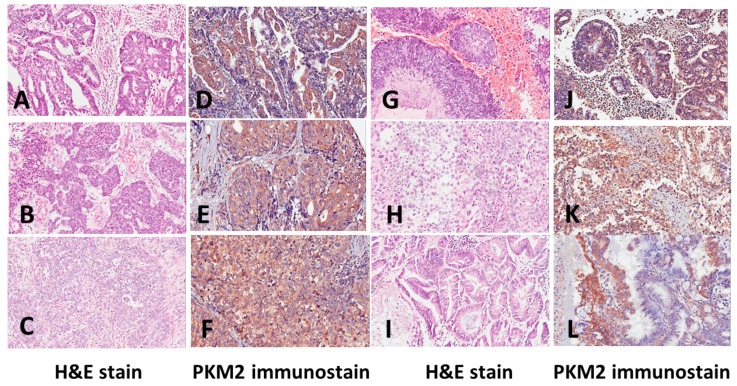
Examples of hematoxylin and eosin (H&E) staining of (**A**) grade 1 endometrioid adenocarcinoma, (**B**) grade 2 endometrioid adenocarcinoma, (**C**) grade 3 endometrioid adenocarcinoma, (**G**) serous carcinoma, (**H**) clear cell carcinoma, and (**I**) mucinous carcinoma and pyruvate kinase M2 (PKM2) immunostaining of (**D**) grade 1 endometrioid adenocarcinoma, (**E**) grade 2 endometrioid adenocarcinoma, (**F**) grade 3 endometrioid adenocarcinoma, (**J**) serous carcinoma, (**K**) clear cell carcinoma, and (**L**) mucinous carcinoma.

We performed immunohistochemistry analyses on endometrial tissue microarrays to evaluate the PKM2 protein expression in normal endometrium and endometrial lesions, including EH without atypia, AH, and EC. PKM2 is mainly present in the cytoplasm. We observed a wide range of variation in the PKM2 levels between the samples. Figure 1 shows examples of normal endometrium (A) and premalignant endometrial lesions of EH without atypia (B) and AH (C), as well as PKM2 protein expression in normal endometrium (D) and premalignant endometrial lesions of EH without atypia (E) and AH (F). Figure 2 shows examples of EC, including G1 of EmAC (A), G2 of EmAC (B), G3 of EmAC (C), SC (G), CC (H), MC (I), and PKM2 protein expression in G1 of EmAC (D), G2 of EmAC (E), G3 of EmAC (F), SC (J), CC (K), and MC (L). The general PKM2 immunoreactivity was significantly lower in normal endometrium and premalignant endometrial lesions (including EH without atypia and AH). In contrast, strong PKM2 immunoreactivity was observed in EC (includes EmAC, SC, CC, and MC).

Semiquantitative analysis of PKM2 immunostaining was performed. As shown in Table 1, PKM2^high^ cells were significantly more abundant in EC (54/108; 50.0%) compared to normal endometrium (0/30; 0.0%), EH without atypia (0/36; 0.0%), and AH (4/32; 12.5%) (*p* < 0.001). PKM2^low^ cells were significantly more abundant in normal endometrium (30/30; 100%), EH without atypia (36/36; 100%), and AH (28/32; 87.5%) compared to EC (54/108; 50.0%) (*p* < 0.001). There were significant differences in both the extent and intensity of PKM2 immunostaining between the hyperplastic and the neoplastic endometrium groups (*p* < 0.001). The PKM2^high^ score was applied as a diagnostic criterion to distinguish between EC, normal endometrium, and premalignant endometrium, which indicated a sensitivity of 50%, a specificity of 95.9%, a positive predictive value (PPV) of 93.1%, and a negative predictive value (NPV) of 66.2%.

Because there were considerable variations in the immunoreactivity of PKM2 in the EC samples, we evaluated whether this discrepancy had any effect on the clinical factors of the affected patients. Twenty three of the 108 EC cases were excluded because of loss to follow-up. The remaining 85 EC cases were classified according to pathology stages using the 2009 FIGO staging system (54 stage I and II, 31 stage III and IV). 

We analyzed the association between the PKM2 immunostaining score and clinicopathological features in patients with EC (n = 85, including 64 EmAC, 11 SC, 8 CC and 2 MC; 41 G1, 19 G2, 25 G3; Table 2). The PKM2 immunostaining score significantly associated with age (*p* = 0.009). Higher PKM2 scores were found among older patients. The PKM2 immunostaining score showed no significant associations with the FIGO stage (*p* = 0.712), nuclear grade (*p* = 0.202), or subtype of EC (*p* = 0.135 for the comparisons of various histological types and *p* = 0.345 for EmAC and non-EmAC comparisons; Table 2).

**Figure 3:**
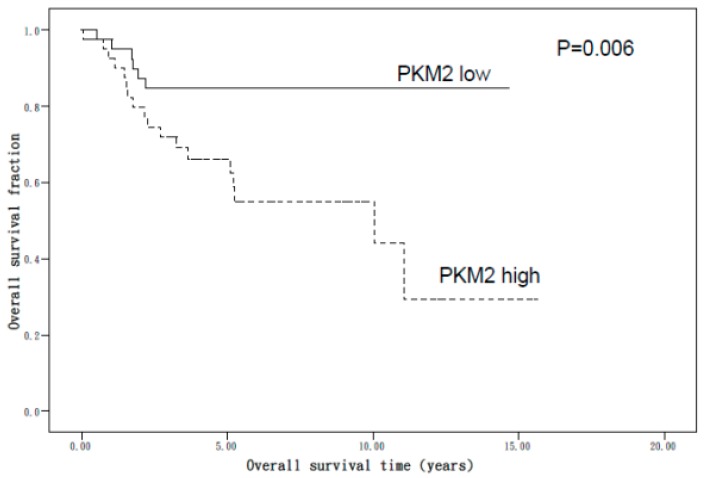
Kaplan–Meier analysis of survival in patients with endometrial carcinoma (EC) based on pyruvate kinase M2 (PKM2) protein immunostaining. Patients with a higher PKM2 score had a poor overall survival compared with those with a lower score in all patients with EC (*p* = 0.006).

The PKM2 immunostaining score’s prognostic value was analyzed in relation to overall survival (OS). Figure 3 shows the results of the Kaplan–Meier survival analysis stratified according to the PKM2 score. Patients who had a higher PKM2 score also had poor OS compared with those who had a lower PKM2 score in cases of EC (*p* = 0.006). The multivariate analysis (Table 3) revealed that higher PKM2 levels conferred a hazard ratio of death of 3.40 (95% confidence interval (CI), 1.35–8.56), higher tumor stage conferred a hazard ratio of death of 8.41 (95% CI, 3.28–21.58), higher nuclear grade 3 conferred a hazard ratio of death of 4.78 (95% CI, 1.79–12.76), and non-EmAC conferred a hazard ratio of death of 2.90 (95% CI, 1.27–6.63). After adjusting for age, stage, and histological grade, the Cox proportional hazards regression analysis revealed a dependent effect of PKM2high on OS, with higher PKM2 levels conferring a hazard ratio of death of 1.96 (95% CI, 0.71–5.37).

## 4. Discussion

The number of cases of EC has been increasing in recent years. The 1994 WHO classification system subdivides EH into EH without atypia and AH. Type 1 EC are estrogen-responsive and preceded by a precursor AH [2]. Cases of AH may be an underdiagnosis of EC, and AH may also be overdiagnosed when epithelial metaplastic changes occur in EH without atypia [5]. Several studies indicate that AH diagnosed through biopsy or curettage is accompanied by EC in 15%–50% of immediate hysterectomy specimens, and some of them are myoinvasive [5,18,19]. Both the underestimation and overestimation of AH severity are quite common. Nevertheless, correct diagnosis of endometrial lesions should be aimed at preventing overdiagnosis and the consequent surgical risks, as well as underdiagnosis and the associated compromise of survival. Therefore, histological analysis of endometrial samples is not reliable on its own for excluding or diagnosing cancers [20,21]. Stage, histopathology, and differentiation grade are currently acknowledged as prognostic factors for EC, but many tumors relapse in an unanticipated way [22].

Molecular diagnostic tools have been suggested as novel methods to identify such critical turning points and to provide a proper treatment plan for patients with endometrial lesions. Such tools could be used for detecting “hidden” EC in patients diagnosed with AH and in the differential diagnosis of premalignant and malignant lesions. There are various genetic alterations in patients with EC, including microsatellite instability and mutations of the phosphatase and tensin homolog (PTEN), K-RAS, and β-catenin genes. In addition, epigenetic changes have been observed, such as the promoter methylation of tumor suppressor genes [23,24,25]. Although potential biomarkers for the prediction of EC are employed, in practice, they may be discordant and ultimately noncontributory [26].

Cancer metabolism provides a key to cancer therapy. Otto Warburg noted aerobic glycolysis in tumors in 1956, and PKM2 is its rate-limiting enzyme [11,27,28]. In recent years, more and more research has established the relationships between oncogenic pathways and tumor metabolism [29]. PKM2 is especially expressed in proliferating cells, such as embryonic stem cells and cancer cells [30]. Recent studies revealed that PKM2 promotes tumorigenesis in gastric cancer and breast cancer [31,32]. Our previous study showed that a PKM2 inhibitor significantly inhibited the glycolytic rate in an ovarian cancer cell line, and as well as attenuation of the extracellular acidification rate [12]. To our knowledge, PKM2’s role in endometrial carcinogenesis remains unclear. Therefore, our interest has been aroused to determine whether the expression of PKM2 in the endometrium could help to improve pathological diagnosis. Upregulation of glycolytic pathway have crucial roles in regulating metabolic changes during carcinogenesis or tumor metastasis [33]. The PKM2 catalyzes the final and also the rate-limiting reaction in the glycolytic pathway [34]. Our findings indicate that high expression of PKM2 is associated with poor prognosis, may be related to metabolic reprogramming in cancer cell progression or tumor metastasis.

A recent study examined PKM1 and PKM2 as metabolic biomarkers to predict the progression of EH to invasive cancer status [35]. The study demonstrated that PKM2 staining could not discriminate between EH and the possibility of progression to invasive cancer. It also demonstrated that lack of PKM1 immunostaining was observed in patients with EH with possibility of progression to EC, it may be through the isoform switch from PKM1 to PKM2 resulted in high PKM2 expression in EC. A previous study has revealed that PKM1 is highly expressed in normal tissues, whereas PKM2 is predominantly expressed in cancer cells [36]. Our study focuses on the examination of PKM2 and endometrial lesions due to PKM2 is a key glycolytic enzyme and is upregulated in multiple human malignancies. It showed that PKM2 expression was higher in AH and especially in EC tissues than in EH and normal control samples. Although the proportion of PKM2 overexpression is not high in AH, but if the patient has the possibility of AH, it needs to be closely follow up and may be choice for surgical candidate. The results suggested that higher PKM2 expression may be a potential warning biomarker for the presence of AH or even EC since it is not easy to discriminate the lesions by H&E staining alone. The metabolic marker may provide another method for evaluating the risk of malignancy.

In summary, this study showed that the levels of PKM2 protein might be useful as a biomarker for the differential diagnosis of malignant and premalignant endometrial lesions. Furthermore, it also seems to be a dependent prognostic factor for patients with EC. Because this study is limited by the sample size, it may be inappropriate to apply the results directly to the whole population. A larger population-based study is warranted to validate the use of PKM2 as a biomarker for the pathological diagnosis of normal endometrium, EH, and EC.

## 5. Conclusions

The results of our study revealed significant differences in both the extent and intensity of PKM2 immunostaining between the hyperplastic and the neoplastic endometrium groups. The PKM2 high score was applied as a diagnostic criterion to distinguish between EC, normal endometrium, and premalignant endometrium. It is also a worse prognostic factor of endometrial carcinoma.

## Figures and Tables

**Table 1:** The Chi-square test for pyruvate kinase M2 (PKM2) expression score based on the slide area and the intensity of color reaction.

	PKM2 ^low^ n. (%)	PKM2 ^high^ n. (%)	*p* Value
			<0.001
Normal	30 (100.0)	0(0)	
EH	36 (100.0)	0(0)	
AH	28 (87.5)	4 (12.5)	
EC	54 (50.0)	54 (50.0)	

Low expression of PKM2 is represented 0+, 1+; high expression of PKM2 is represented 2+, 3+, 4+, 6+. Normal = normal endometrium; EH = endometrial hyperplasia without atypia; AH = atypical hyperplasia; EC = endometrial carcinoma.

**Table 2:** Clinicopathological features in 85 patients.

	PKM2 Expression Low	PKM2 Expression High	*p* Value
Characteristic	0+, 1+	2+, 3+, 4+, 6+	
Patients (no.)	42	43	
Age (years)			0.009
Range	21–81	32–89	
Mean ± SEM	52.21 ± 1.60	58.77 ± 1.85	
FIGO Stage [no. (%)]			0.712
I, II	28 (51.9)	26 (48.1)	
III, IV	14 (45.2)	17 (54.8)	
Nuclear grade [no. (%)]			
G1	24 (58.5)	17 (41.5)	0.202
G2	9 (47.4)	10 (52.6)	
G3	9 (36.0)	16 (64.0)	
Histological type [no. (%)]			0.135 ^a^
CC	1 (12.5)	7 (87.5)	
EmAC	34 (53.1)	30 (46.9)	
MC	1 (50.0)	1 (50)	
SC	6 (54.5)	5 (45.5)	
Histological type [no. (%)]			0.345
EmAC	34 (53.1)	30 (46.9)	
Non- EmAC	8 (38.1)	13 (61.9)	

^a^ Fisher’s exact test. SEM = standard error of the mean; G1 = nuclear grade 1; G2 = nuclear grade 2; G3 = nuclear grade 3; EmAC = endometrioid adenocarcinoma; CC = clear cell carcinoma; MC = mucinous carcinoma; SC = serous carcinoma; PKM2 = Pyruvate kinase M2.

**Table 3:** Multivariate survival analysis of clinicopathological factors in 85 patients.

Variable	Univariate Analysis Crude HR (95% CI)	Multivariate Adjusted HR (95% CI)
Age (years)	1.04 (1.00–1.07) *	1.04 (1.00–1.08)
PKM2 expression ^a^		
Low	1.00 (Ref.)	1.00 (Ref.)
High	3.40 (1.35–8.56) *	1.96 (0.71–5.37)
FIGO Stage		
I, II	1.00 (Ref.)	1.00 (Ref.)
III, IV	8.41 (3.28–21.58) *	7.97 (2.71–23.48) *
Nuclear grade		
G1	1.00 (Ref.)	1.00 (Ref.)
G2	1.54 (0.47–5.07)	0.89 (0.24–3.28)
G3	4.78 (1.79–12.76) *	4.04 (0.96–16.99)
Histological type		
EmAC	1.00 (Ref.)	1.00 (Ref.)
Non-EmAC	2.90 (1.27–6.63) *	0.32 (0.08–1.30)

* *p* < 0.05. CI = confidence interval; HR = hazard ratio; Ref = reference group; G1 = nuclear grade 1; G2 = nuclear grade 2; G3 = nuclear grade 3; EmAC = endometrioid adenocarcinoma ^a^ Low expression of PKM2 is represented 0+, 1+; high expression of PKM2 is represented 2+, 3+, 4+, 6+.

## References

[1] Emons G., Fleckenstein G., Hinney B., Huschmand A., Heyl W. (2000). Hormonal interactions in endometrial cancer. Endocr. Relat. Cancer.

[2] Kurman R.J., Kaminski P.F., Norris H.J. (1985). The behavior of endometrial hyperplasia. A long-term study of “untreated” hyperplasia in 170 patients. Cancer.

[3] Mutter G.L. (2000). Histopathology of genetically defined endometrial precancers. Int. J. Gynecol. Pathol..

[4] Mutter G.L. (2002). Diagnosis of premalignant endometrial disease. J. Clin. Pathol..

[5] Silverberg S.G. (2000). Problems in the Differential Diagnosis of Endometrial Hyperplasia and Carcinoma. Mod. Pathol..

[6] Sanderson P.A., Critchley H.O., Williams A.R., Arends M.J., Saunders P.T. (2017). New concepts for an old problem: The diagnosis of endometrial hyperplasia. Hum. Reprod. Update.

[7] Hanahan D., Weinberg R.A. (2011). Hallmarks of Cancer: The Next Generation. Cell.

[8] Pavlova N.N., Thompson C.B. (2016). The Emerging Hallmarks of Cancer Metabolism. Cell Metab..

[9] Noguchi T., Inoue H., Tanaka T. (1986). The M1- and M2-type isozymes of rat pyruvate kinase are produced from the same gene by alternative RNA splicing. J. Boil. Chem..

[10] Christofk H.R., Vander Heiden M.G., Harris M.H., Ramanathan A., Gerszten R.E., Wei R., Fleming M.D., Schreiber S.L., Cantley L.C. (2008). The M2 splice isoform of pyruvate kinase is important for cancer metabolism and tumour growth. Nature.

[11] Anastasiou D., Yu Y., Israelsen W.J., Jiang J.K., Boxer M.B., Hong B.S., Tempel W., Dimov S., Shen M., Jha A. (2012). Pyruvate kinase M2 activators promote tetramer formation and suppress tumorigenesis. Nat. Chem. Biol..

[12] Chao T.-K., Huang T.-S., Liao Y.-P., Huang R.-L., Su P.-H., Shen H.-Y., Lai H.-C., Wang Y.-C. (2017). Pyruvate kinase M2 is a poor prognostic marker of and a therapeutic target in ovarian cancer. PLoS ONE.

[13] Hidalgo A., Piña P., Guerrero G., Lazos M., Salcedo M. (2003). A simple method for the construction of small format tissue arrays. J. Clin. Pathol..

[14] Scully R.E., Bonfiglio T.A., Kurman R.J., Silverberg S.G., Wilkinson E.J. (1994). Uterine Corpus. Histological Typing of Female Genital Tract Tumours.

[15] Pecorelli S. (2009). Revised FIGO staging for carcinoma of the vulva, cervix, and endometrium. Int. J. Gynecol. Obstet..

[16] Jin J.-S., Hsieh D.-S., Loh S.-H., Chen A., Yao C.-W., Yen C.-Y. (2006). Increasing expression of serine protease matriptase in ovarian tumors: Tissue microarray analysis of immunostaining score with clinicopathological parameters. Mod. Pathol..

[17] Sarmadi S., Izadi-Mood N., Sotoudeh K., Tavangar S.M. (2009). Altered PTEN expression; a diagnostic marker for differentiating normal, hyperplastic and neoplastic endometrium. Diagn. Pathol..

[18] Allison K.H., Reed S.D., Voigt L.F., Jordan C.D., Newton K.M., Garcia R.L. (2008). Diagnosing endometrial hyperplasia: Why is it so difficult to agree?. Am. J. Surg. Pathol..

[19] Kendall B.S., Ronnett B.M., Isacson C., Cho K.R., Hedrick L., Diener-West M., Kurman R.J. (1998). Reproducibility of the diagnosis of endometrial hyperplasia, atypical hyperplasia, and well-differentiated carcinoma. Am. J. Surg. Pathol..

[20] Suh-Burgmann E., Hung Y.Y., Armstrong M.A. (2009). Complex atypical endometrial hyperplasia: The risk of unrecognized adenocarcinoma and value of preoperative dilation and curettage. Obstet. Gynecol..

[21] Taylor P.J., Gomel V. (1995). 2 Endometrial ablation: Indications and preliminary diagnostic hysteroscopy. Baillière’s Clin. Obstet. Gynaecol..

[22] Teng Y., Ai Z., Wang Y., Wang J., Luo L. (2013). Proteomic identification of PKM2 and HSPA5 as potential biomarkers for predicting high-risk endometrial carcinoma. J. Obstet. Gynaecol. Res..

[23] Matias-Guiu X., Catasus L., Bussaglia E., Lagarda H., Garcia A., Pons C., Muñoz J., Argüelles R., Machin P., Prat J. (2001). Molecular pathology of endometrial hyperplasia and carcinoma. Hum. Pathol..

[24] Hecht J.L., Mutter G.L. (2006). Molecular and Pathologic Aspects of Endometrial Carcinogenesis. J. Clin. Oncol..

[25] Horn L.-C., Meinel A., Handzel R., Einenkel J. (2007). Histopathology of endometrial hyperplasia and endometrial carcinoma. Ann. Diagn. Pathol..

[26] Banno K., Kisu I., Yanokura M., Tsuji K., Masuda K., Ueki A., Kobayashi Y., Yamagami W., Nomura H., Tominaga E. (2012). Biomarkers in endometrial cancer: Possible clinical applications (Review). Oncol. Lett..

[27] Vander Heiden M.G., Cantley L.C., Thompson C.B. (2009). Understanding the Warburg effect: The metabolic requirements of cell proliferation. Science.

[28] Christofk H.R., Vander Heiden M.G., Wu N., Asara J.M., Cantley L.C. (2008). Pyruvate kinase M2 is a phosphotyrosine-binding protein. Nature.

[29] Liu L., Ulbrich J., Müller J., Wüstefeld T., Aeberhard L., Kress T.R., Muthalagu N., Rycak L., Rudalska R., Moll R. (2012). Deregulated MYC expression induces dependence upon AMPK-related kinase 5. Nature.

[30] Lee J., Kim H.K., Han Y.-M., Kim J. (2008). Pyruvate kinase isozyme type M2 (PKM2) interacts and cooperates with Oct-4 in regulating transcription. Int. J. Biochem. Cell Boil..

[31] Lim J.Y., Yoon S.O., Seol S.Y., Hong S.W., Kim J.W., Choi S.H., Cho J.Y. (2012). Overexpression of the M2 isoform of pyruvate kinase is an adverse prognostic factor for signet ring cell gastric cancer. World J. Gastroenterol..

[32] Li W., Liu J., Jackson K., Shi R., Zhao Y. (2014). Sensitizing the Therapeutic Efficacy of Taxol with Shikonin in Human Breast Cancer Cells. PLoS ONE.

[33] Cheng T.Y., Yang Y.C., Wang H.P., Tien Y.W., Shun C.T., Huang H.Y., Hsiao M., Hua K.T. (2018). Pyruvate kinase M2 promotes pancreatic ductal adenocarcinoma invasion and metastasis through phosphorylation and stabilization of PAK2 protein. Oncogene.

[34] Wong N., De Melo J., Tang D. (2013). PKM2, a Central Point of Regulation in Cancer Metabolism. Int. J. Cell Boil..

[35] Hosseini Nasab S., Jooya N., Esmaeili A., Zarrin Khameh N., Diaz-Arrastia C., Momeni M. (2018). Using Pyruvate Kinase as a Predictor for Patient with Endometrial Cancer Having Complex Hyperplasia with Atypia to Prevent Hysterectomy and Preserve Fertility: Retrospective Immunohistochemical Study. Reprod. Sci..

[36] Yang W., Lu Z. (2015). Pyruvate kinase M2 at a glance. J. Cell Sci..

